# Momentary Associations Between Emotional Responses to Social Media and Affect: Consistency Across Global Affect and Specific Emotional States

**DOI:** 10.1007/s42761-024-00257-x

**Published:** 2024-08-22

**Authors:** Simone Imani Boyd, Melissa J. Dreier, Saskia L. Jorgensen, Serena L. Moghaddas, Evan Kleiman, Jessica L. Hamilton

**Affiliations:** 1 Department of Psychology, Rutgers, The State University of New Jersey, Tillet Hall, 53 Avenue East, Piscataway, NJ 08854 USA; 2 Department of Psychology, George Washington University, Washington, D.C USA; 3 GenPsych LLC, Princeton, NJ USA

**Keywords:** Positive affect, Negative affect, Social media, Adolescents, Ecological momentary assessment

## Abstract

Social media (SM) is widely used among adolescents, making the relationship between SM and affect in this population important to understand. However, previous research has largely used methods that do not capture the dynamic and variable nature of SM or measure the consistency of effects across global affect and specific emotions. The current study examined the momentary relationship between positive and negative SM experiences and global positive affect (PA) and negative affect (NA), as well as specific affective states of PA and NA. Adolescents (*N* = 62; 14–17 years old) were recruited from SM platforms to participate in an eight-week ecological momentary assessment study. Participants reported three times per day on PA and NA and positive and negative SM experiences. Random intercept and random slope models were used to examine within-person associations between momentary positive and negative SM experiences and affect after controlling for between-person differences. At prompts when participants reported greater-than-usual positive SM experiences, they reported higher global PA and lower global NA. At prompts when participants reported greater than their average negative SM experiences, they endorsed lower global PA and higher global NA. These results were consistent across same-valence specific affective states. The current study highlights proximal associations between SM experiences and affect, highlighting potential avenues of short-term risk and protection.

**Supplementary Information:**

The online version contains supplementary material available at 10.1007/s42761-024-00257-x.

Social media (SM) use is nearly ubiquitous among adolescents in the USA; nearly all adolescents use some form of SM and 46% report being online “almost constantly” (Vogels et al., 2022). SM provides adolescents with opportunities to connect and interact with peers, broader networks, and information that can mirror, extend, and transform offline experiences and interactions (Hamilton, Dreier et al., 2023). The unique features of SM use (Nesi et al., 2018a, b) in combination with the developmental context of adolescence have been demonstrated to create windows of sensitivity within this developmental period for the effects of SM use on well-being (Orben et al., 2022). Despite the variety of SM experiences, evidence regarding the association between SM use and affective disorders is largely derived from research on the *frequency* or *duration* of SM use, yielding small or negligible effects (Odgers & Jensen, 2020; Orben & Przybylski, 2019). An emerging body of research investigating SM experiences has identified key nuances in the relationship between SM use and affect that warrant further attention.

## Social Media Experiences and Affect

Cross-sectional and longitudinal studies have linked negative SM experiences to depression and loneliness (Bonsaksen et al., 2023; Magis-Weinberg et al., 2021). Specifically, negative SM events such as cyberbullying (Nixon, 2014), peer exclusion or fear of missing out (Marengo et al., 2021), and negative social comparison (Nesi & Prinstein, 2015; Peters et al., 2021) are associated with NA. However, less research has examined the associations between negative experiences and PA, or positive experiences and either NA or PA. An experimental study found that adolescents randomly assigned to receive fewer “likes” and less positive feedback in a SM paradigm reported higher NA than those who received more likes or feedback (Lee et al., 2020). Meta-analytic and longitudinal studies also have demonstrated that positive aspects of SM use, like online disclosure between friends, one-to-one communication, or funny online experiences, buffer against loneliness (Magis-Weinberg et al., 2021; Marciano et al., 2022). However, Nesi and colleagues (2021) found that positive and negative emotional responses to SM were cross-sectionally associated with depressive symptoms, whereas only positive emotional responses to SM prospectively predicted increased depressive symptoms one year later (Nesi et al., 2021). Despite the importance of longitudinal studies of SM experiences and affect, they do not capture the proximal effects of SM experiences on affect.

### Social Media Use and Affect as Dynamic Processes

Important methodological shortcomings of previous studies limit our understanding of the association between SM experiences and affect. First, the intensity and duration of affective states differ not only between individuals, but also when comparing an individual to themselves over time (Kuppens et al., 2022; Zelenski & Larsen, 2000). Affective variability has been demonstrated to unfold over brief time scales within-person (Kuppens, 2015; Kuppens et al., 2010). Likewise, SM use and one’s experiences can fluctuate over brief periods of time (Hamilton, Hutchinson et al., 2023) and these fluctuations have been linked to changes in sleep (Hamilton et al., 2020), loneliness (Silva et al., 2023), and social connectedness (Armstrong-Carter et al., 2023). However, there is a lack of research examining whether fluctuations in SM experiences predict changes in affect, particularly while capturing the dynamic nature of both constructs. Given the fluctuating nature of both SM and affect, methodological and statistical approaches like ecological momentary assessment (EMA) and multilevel modeling are uniquely suited to disentangle the associations between SM experiences and affect. Furthermore, the use of EMA strengthens ecological validity because variables of interest are measured in participants’ natural setting, reducing participants’ retrospective bias.

### Specific Emotions

Research on SM and affect mainly focuses on global affect (overall NA or PA), which does not take into account the depth and breadth of emotional experiences characteristic of adolescence. In particular, adolescence is an important period for emotional development. Adolescents begin to cultivate the ability to experience, identify, and independently regulate specific emotions (Nook & Somerville, 2019). Moreover, the within-person factor structure of affect resembles the structure of specific emotional states rather than global affective states (Jacobson et al., 2023). SM provides a diversity of experiences, and specific experiences may differentially elicit distinct positive and negative emotional states (Ellsworth & Smith, 1988; Lazarus & Smith, 1988). For example, becoming aware that one has been excluded from a social gathering may elicit feelings of loneliness more so than anger or worry. Understanding the consistency of SM effects across global affect and specific emotional states can elucidate the experiences underlying global and specific affect dynamics implicated in the presentation and treatment of affective disorders (Bosley et al., 2019; Schoevers et al., 2021). However, specific emotional responses to SM may be obscured by examining only global NA/PA or single emotional states.

### Current Study

The current study investigates the momentary relationship between positive and negative SM experiences and affect among adolescents, including at the global level (e.g., overall PA and NA) and specific emotional states (e.g., satisfaction, hopefulness, worry, and irritability). It was hypothesized that after controlling for between-person differences in positive and negative SM experiences, momentary increases in negative SM experiences would be associated with decreased momentary global PA and increased momentary global NA. It was hypothesized that increases in momentary positive SM experiences would be associated with increased momentary global PA and decreased momentary global NA. Finally, given limited prior research examining specific affective states, we examined the within-person associations between positive and negative SM experiences and specific positive and negative emotions and compared these associations to the patterns of effects for respective global affective states.

## Method

### Recruitment and Procedure

Participants were recruited through ad campaigns on Instagram and TikTok to participate in an eight-week intensive monitoring study. Participants in the current sample were recruited to participate between January 2022 and August 2023. The objective of this study was to understand the association between SM use and mental health in adolescents. Eligible study participants were between 14 and 17 years old, attended high school (grades 9–12), lived in the USA, were fluent in English to complete study procedures, and used SM on an Android smartphone due to larger study goals and procedures (where certain applications were only compatible with Android phones). All study procedures occurred entirely remotely and online, which facilitated study participation from individuals with diverse backgrounds and geographic locations in the USA. The study was approved by the Institutional Review Board (IRB) at Rutgers University - New Brunswick.

### Procedure

Eligible participants and their parent/legal guardian met with the study team to learn about the study and sign the online consent/assent forms via phone call or video call (Zoom). Participants then met with the study team via Zoom to install study applications, which included the EMA application MetricWire. Participants were emailed the secure link to the baseline survey via Qualtrics, which included demographic questionnaires (e.g., age, gender identity, sexual orientation, racial/ethnic identity, perceived socioeconomic status), as well as questions about their SM use and mental health. Participants then completed eight weeks of an intensive monitoring protocol, which included EMA. Participants received gift card compensation at baseline and the end of the EMA protocol based upon adherence.

#### EMA Protocol

The EMA protocol was completed through MetricWire, which is free to download on all smartphones. Participants were sent push notifications to complete three brief surveys per day in the morning, afternoon, and evening. During the first study meeting, participants selected their preferred times for the morning (weekend and weekday; 6:00 am–11:00 am) and evening surveys (8:00 pm–11:00 pm). Midday surveys were randomly sent between the hours of 3:00 pm and 6:00 pm on weekdays and between the hours of 1:00 pm and 6:00 pm on weekends. Participants had 60 min to complete morning or midday surveys and 90 min to complete evening surveys. Participants were sent automatic reminder notifications 30 min before the survey closed if not yet completed. MetricWire automatically adjusted to send surveys on these schedules in the participant’s time zone.

#### Demographic Questionnaire

At the baseline assessment, participants completed questions about their age, assigned sex at birth, gender identity, sexual orientation, race, and ethnicity. Socioeconomic status (SES) was assessed using the MacArthur Subjective Social Status Scale—Youth Version (Goodman et al., 2001). Participants rated the perceived SES of their family and their schools with responses ranging from 1 (worst off) to 10 (best off).

### EMA Measures

#### Positive and Negative Affect Scale (PANAS)—Short Form

A shortened version of the Positive and Negative Affect Scale (PANAS) with 6 items was included to assess affect in the current study, including 3 positive (happy, satisfied, hopeful) and 3 negative (worried, lonely, irritable) items. Participants were asked to respond on a scale of 0 (not at all) to 4 (extremely) about their emotional state (e.g., “Please rate how much you are feeling happy right now”). Participants responded to the PANAS at all three daily timepoints (morning, afternoon, and evening). In the present study, affective states were summed to create total positive affect (PA) and negative affect (NA) scores to reflect global negative and positive affect. Specific emotions were examined using individual items from the PANAS. The PANAS is a commonly used measure with high validity and reliability and has been previously used in EMA research (Silk et al., 2011).

#### Emotional Responses to Social Media

An adapted version of the Emotional Responses to SM (ERSM) Scale (Nesi et al., 2021) assessed participant’s emotional reactions to experiences or events that occurred on SM in the morning, afternoon, and evening EMA surveys. During all EMA surveys, participants were asked to respond on a scale of 0 (not at all) to 6 (extremely) when thinking about their most recent SM use. In the present study, seven items based on the ERSM were used, including 4 negative emotional responses (e.g., “Thinking about the last time you used SM, how much did you feel: worried that you are missing out on things?”) and 3 positive emotional responses (e.g., “Thinking about the last time you used SM, how much did you feel supported or encouraged by others?”). The three positive items were averaged within each prompt to create momentary positive SM experience scores. The four negative items were averaged within each prompt to create momentary negative SM experience scores.

### Analytic Approach

Data preparation and analysis was conducted in R using tidyverse (Wickham et al., 2019) and lme4 (Bates et al., 2014). Intraclass correlations were calculated to determine the amount of variability in predictor variables that could be attributed to between- vs. within-person variability. Multilevel modeling with random intercepts and random slopes was conducted to investigate the association between momentary fluctuations in SM experiences and momentary affect. The models presented included Level-1 (or within-person) predictors and Level-2 (or between-person) predictors. Level-1 predictors were the predictors of interest, including momentary SM experiences, whereas Level-2 predictors were incorporated as covariates. Between-person (Level-2) means of positive and negative SM experiences were included for each participant, to account for between-person differences in higher or lower average SM experiences. Subsequently, momentary ratings of emotional responses to SM experiences were person-centered to represent within-person momentary fluctuations in SM experiences compared to a person’s average or mean across the study using EMAtools (Kleiman, 2021). For example, a person-centered score for a participant’s morning positive emotional response to SM experiences on a given day reflects higher levels of positive responses compared to the person’s average across the study. These person-centered scores reflect individual momentary fluctuations within a person (Level-1) in SM experiences. Each model contained two Level-1 variables (person-centered positive SM experiences and person-centered negative SM experiences) and two Level-2 variables (person-mean positive SM experiences and person-mean negative SM experiences). In total, eight models were conducted within the current study.

Random intercepts and slopes were incorporated into the models to reflect individual differences in affect and in the association between person-centered emotional responses to SM experiences and affect between participants. Likelihood ratio tests were conducted to determine if the inclusion of random slopes benefitted the model. Between- and within-person associations between emotional responses to SM experiences and global positive and negative affect were examined first. Then, specific emotional states were examined in contrast to the patterns of results of their respective global affect (e.g., happiness and global PA).

## Results

### Descriptive Results

The sample included 62 adolescents (14–17 years old; *M* = 16.15; *SD* = 0.97) who completed the baseline demographic measures and EMA portion of the study. Of the 62 participants, 30 (49.2%) identified as girls, 10 (16.4%) identified as boys, 21 (34.4%) identified as genderqueer, and 1 participant chose not to disclose their gender identity. In terms of racial/ethnic identity, 25 (40.3%) participants identified as White non-Hispanic/Latine, 13 (21.0%) participants identified as Black/African American, 6 (9.7%) participants identified as Asian/Pacific Islander, 8 (12.9%) identified as Biracial or Multiracial, and 10 (16.1%) identified as Hispanic/Latine. In regard to sexual orientation, 39 (62.9%) participants identified as LGBQ + . The majority of participants were in grades 10 (26.2%), 11 (31.1%), and 12 (32.8%). Participants’ perceived family and school socioeconomic status represented the full range of possible responses (1–10); however, on average participants endorsed that their family (*M* = 5.23, *SD* = 1.77) and school (*M* = 5.05, *SD* = 2.04) were in the middle-class range.

The results come from 2,482 morning surveys, 2,393 midday surveys, and 2,700 evening surveys completed by participants. Of the possible days for survey completion that contained both the ERSM items and the PANAS items (*N* = 56), participants completed 75.37% of the morning surveys (1.79–100%; *M* = 43.62 days, *SD* = 10.51), 78.39% of the midday surveys (1.79–100%; *M* = 43.90 days, *SD* = 10.93), and 80.94% of evening surveys (3.57–100%; *M* = 45.33 days, *SD* = 10.27). Means, standard deviations, and bivariate correlations between the central model variables are presented in Table 1. Intraclass correlations of predictor and outcome variables are presented in Table 2. The variability in positive and negative SM experiences, global PA, hopefulness, and loneliness was explained more by between- than within-person differences. In contrast, the variability in happiness, satisfaction, global NA, worry, and irritability was explained more by within- than between-person differences.

**Table 1:** Bivariate correlations

Variable	*M*	*SD*	1	2	3	4	5	6	7	8	9	10	11
1. Global PA	5.54	3.14											
2. Global NA	3.60	2.91	− .28**										
3. Happiness	2.09	1.11	.87**	− .27**									
4. Satisfaction	1.74	1.22	.89**	− .30**	.68**								
5. Hopefulness	1.72	1.24	.88**	− .17**	.64**	.66**							
6. Worry	1.35	1.26	− .18**	.79**	− .16**	− .23**	− .10**						
7. Irritability	1.07	1.16	− .28**	.78**	− .30**	− .27**	− .18**	.46**					
8. Loneliness	1.19	1.30	− .20**	.78**	− .19**	− .21**	− .13**	.39**	.41**				
9. PM positive SM experiences	2.04	1.19	.41**	.15**	.37**	.33**	.38**	.13**	.08**	.14**			
10. PC positive SM experiences	.00	1.01	.20**	− .08**	.19**	.17**	.16**	− .06**	− .08**	− .06**	.00		
11. PM negative SM experiences	1.13	.94	.05**	.43**	.06**	.01	.07**	.33**	.28**	.39**	.39**	.00	
12. PC negative SM experiences	.00	.76	− .07**	.20**	− .06**	− .09**	− .05**	.16**	.13**	.18**	.00	.13**	.00

*PM* person-mean (between-person), *PC* person-centered (within-person)

*p < .05; **p < .01

**Table 2:** Intraclass correlations

	Intraclass correlation
Positive emotional responses to SM	.61
Negative emotional responses to SM	.61
Global positive affect	.54
*Happiness*	.41
*Satisfaction*	.41
*Hopefulness*	.53
Global negative affect	.49
*Worry*	.37
*Irritability*	.37
*Loneliness*	.54

### Emotional Responses to Social Media Experiences and Global Positive Affect

Fixed- and random-slope models with positive and negative SM experiences predicting global PA were compared via likelihood ratio test, which indicated that the random-slope model was a better fitting model than the fixed-slope model (χ^2^(5) = 273.33, *p* < .001). Results for this model are presented in Table 3. At the within-person level, there were significant associations between global PA and positive SM experiences (*B* = .67, *p* < .001), as well as negative SM experiences (*B* =  − .43, *p* < .001). At prompts when participants endorsed a higher level than their average positive SM experiences, they also had higher global PA. In contrast, at prompts when participants endorsed a higher level than their average negative SM experiences, they also had lower global PA. There was a significant association at the between-person level between global PA and positive SM experiences (*B* = 1.18, *p* < .001). Participants with higher average positive SM experiences also had higher global PA. However, there was not a significant association between global PA and negative SM experiences at the between-person level (*B* =  − .37, *p* = .10).

**Table 3:** Multilevel models of global PA and specific positive emotional states

	*B*	*SE*	*p*
*Global positive affect*			
Person-mean negative ERSM	− .37	.22	.10
Person-centered negative ERSM	− .43	.08	< .001
Person-mean positive ERSM	1.18	.17	< .001
Person-centered positive ERSM	.67	.05	< .001
*Happiness*			
Person-mean negative ERSM	− .11	.07	.09
Person-centered negative ERSM	− .12	.03	< .001
Person-mean positive ERSM	.35	.05	< .001
Person-centered positive ERSM	.23	.02	< .001
*Satisfaction*			
Person-mean negative ERSM	− .19	.08	.02
Person-centered negative ERSM	− .19	.03	< .001
Person-mean positive ERSM	.41	.06	< .001
Person-centered positive ERSM	.23	.02	< .001
*Hopefulness*			
Person-mean negative ERSM	− .08	.10	.43
Person-centered negative ERSM	− .12	.03	< .001
Person-mean positive ERSM	.42	.07	< .001
Person-centered positive ERSM	.21	.02	< .001

### Emotional Responses to Social Media Experiences and Global Negative Affect

Similar to global PA, fixed- and random-slope models with positive and negative SM experiences predicting global NA were compared via likelihood ratio test which indicated that the random-slope model fit the data better than the fixed slope model (χ^2^(5) = 153.25, *p* < .001). At the within-person level, global NA was significantly associated with both positive and negative SM experiences (*B* =  − .37, *p* < .001; *B* = .80, *p* < .001, respectively). During prompts when a participant endorsed a greater level than their average positive SM experiences, they also endorsed lower global NA. Conversely, during prompts when a participant endorsed a greater level than their average negative SM experiences, they also endorsed greater global NA. At the between-person level, negative SM experiences were significantly associated with global NA (*B* = 1.42, *p* < .001). Participants with higher average negative SM experiences throughout the study period also endorsed higher levels of global NA. However, there were no significant associations between positive SM experiences and global NA at the between-person level (*B* =  − .19, *p* = .26).

### Emotional Responses to Social Media Experiences and Specific Positive Emotions

Fixed- and random-slope models were compared using likelihood ratio tests with positive and negative SM experiences predicting happiness, satisfaction, and hopefulness. Random-slope models were indicated as better fitting for models predicting happiness (χ^2^(5) = 211.69, *p* < .001), satisfaction (χ^2^(5) = 146.57, *p* < .001), and hopefulness (χ^2^(5) = 188.18, *p* < .001). The pattern of within-person results for specific positive emotions was similar to those for global PA (Table 3). At prompts when participants had more than their average positive SM experiences, they also endorsed greater happiness (*B* = .23, *p* < .001), satisfaction (*B* = .23, *p* < .001), and hopefulness (*B* = .21, *p* < .001). Moreover, at prompts when participants endorsed more than their average negative experiences, they also endorsed lower happiness (*B* =  − .12, *p* < .001), satisfaction (*B* =  − .19, *p* < .001), and hopefulness (*B* =  − .12, *p* < .001). Similar to global PA, participants with higher mean positive SM experiences also had higher mean happiness (*B* = .35, *p* < .001), satisfaction (*B* = .41, *p* < .001), and hopefulness (*B* = .42, *p* < .001) at the between-person level. Negative SM experiences were not significantly associated with happiness (*B* =  − .11, *p* = .09) or hopefulness (*B* =  − .08, *p* = .43) at the between-person level. However, participants with higher average negative SM experiences had lower average satisfaction (*B* =  − .19, *p* = .02).

### Emotional Responses to Social Media Experiences and Specific Negative Emotions

Fixed- and random-slope models were compared via likelihood ratio tests with positive and negative SM experiences predicting worry, irritability, and loneliness. Random-slope models were indicated to be better fitting for models predicting worry (χ^2^(5) = 163.87, *p* < .001), irritability (χ^2^(5) = 78.79, *p* < .001), and loneliness (χ^2^(5) = 183.53, *p* < .001). The pattern of within-person level results was similar to global NA. At prompts when adolescents had a greater level than their average positive SM experiences, they endorsed reductions in worry (*B* =  − .12, *p* < .001), irritability (*B* =  − .12, *p* < .001), and loneliness (*B* =  − .12, *p* < .001). At prompts when adolescents endorsed more than their mean level of negative SM experiences, they experienced increases in worry (*B* = .27, *p* < .001), irritability (*B* = .21, *p* < .001), and loneliness (*B* = .33, *p* < .001). Similarly, the pattern of results for specific NA emotions was similar to the results for global NA. Participants who had higher negative SM experiences endorsed higher levels of worry (*B* = .45, *p* < .001), irritability (*B* = .41, *p* < .001), and loneliness (*B* = .57, *p* < .001) throughout the study period. Positive SM experiences were not associated with worry (*B* =  − .07, *p* = .27), irritability (*B* =  − .08, *p* = .19), or loneliness (*B* =  − .03, *p* = .66) at the between-person level (Table 4).

**Table 4:** Multilevel models of global NA and specific negative emotional states

	*B*	*SE*	*p*
*Global negative affect*			
Person-mean negative ERSM	1.42	.22	< .001
Person-centered negative ERSM	.80	.06	< .001
Person-mean positive ERSM	− .19	.16	.26
Person-centered positive ERSM	− .37	.04	< .001
*Worry*			
Person-mean negative ERSM	.45	.08	< .001
Person-centered negative ERSM	.27	.03	< .001
Person-mean positive ERSM	− .07	.06	.27
Person-centered positive ERSM	− .12	.02	< .001
*Irritability*			
Person-mean negative ERSM	.41	.08	< .001
Person-centered negative ERSM	.21	.03	< .001
Person-mean positive ERSM	− .08	.06	.19
Person-centered positive ERSM	− .12	.02	< .001
*Loneliness*			
Person-mean negative ERSM	.57	.11	< .001
Person-centered negative ERSM	.33	.03	< .001
Person-mean positive ERSM	− .03	.08	.66
Person-centered positive ERSM	− .12	.02	< .001

## Discussion

The current study examined adolescents’ momentary associations between positive and negative SM experiences and affect, including global NA and PA and specific emotions. Findings demonstrate that when adolescents have greater-than-usual positive SM experiences, they also endorse higher global PA and happiness, satisfaction, and hopefulness, as well as lower global NA and worry, irritability, and loneliness. Conversely, when they experienced greater-than-usual negative responses to SM, they endorsed greater global NA and worry, irritability, and loneliness, as well as lower global PA and happiness, satisfaction, and hopefulness. These findings highlight that SM experiences are associated with adolescents’ broader affective experiences, including specific emotions within NA and PA.

Of note, this study was the first study to examine SM experiences and affect on a proximal (momentary) timescale, which stand in contrast to longitudinal findings demonstrating that, one year apart, positive responses to SM experiences predict depressive symptoms (Nesi et al., 2021). In this study, momentary increases in positive responses to SM compared to a person’s average were associated with greater positive emotions and lower negative emotions at the within-person level. The inverse was the case for increases in negative responses to experiences. In other words, no specific emotions appeared to be driving the broad relationships between SM experiences and affect, which may be due to adolescents’ continued development of affective state differentiation (Nook, 2021). At the same time, given positive and negative SM experiences were aggregated for our study, it is also possible that certain SM experiences are associated with more specific emotions (e.g., irritability), however, our study was not adequately powered to detect this. It also is possible that different SM experiences may affect specific positive and negative emotions for different adolescents, consistent with growing research demonstrating the heterogeneity in adolescents’ experiences and emotional reactions to SM, both between- and within-person (Beyens et al., 2020, 2021; Pouwels et al., 2021; Valkenburg et al., 2021). However, future research is needed to examine these constructs at the person-specific (*N* = 1) level to better understand the nuances of these relationships.

Overall, this study highlights that adolescents’ experiences on SM —just as in-person—impact their broader affective states. Previous longitudinal and meta-analytic studies have found similar affect-modulating properties of SM experiences (Magis-Weinberg et al., 2021; Marciano et al., 2022). The current study extends this research to short-term timeframes, offering important implications for interventions for emotion regulation in response to SM experiences (Wadley et al., 2020). Moreover, a greater awareness of when SM experiences generate negative or positive emotions may allow adolescents to engage in problem-solving or identify specific behavioral strategies to cope with distinct emotional experiences (Linehan, 1993). Although this study provides preliminary support that SM and mood are associated, future research should probe whether SM use may indeed cause changes in mood states and whether SM use can facilitate engagement in evidence-based emotion regulation and coping strategies. Additionally, this study lays the groundwork for future research to test whether SM experiences may be associated with emotion granularity. Future studies with a greater sample size and more observations may test, for example, whether SM experiences moderate the relationship between specific emotions and emotional experience or whether SM experiences are associated with within-person emotion fluctuation.

### Strengths and Limitations

This study had several strengths. Our study data included two months of intensive longitudinal data on adolescents’ SM experiences, which permitted the capture and examination of fluctuations in adolescents’ SM use and affect. The diverse sample including nearly 60% of participants from minoritized racial/ethnic backgrounds and 63% LGBQ + adolescents increases the generalizability of these findings and fills an important gap in a large body of research focusing mostly on homogenous (and mostly non-minoritized) youth. Finally, a strong analytic approach, examining within-person and person-centered relationships, while controlling for between-person associations, allowed this study to disentangle individuals’ unique within-person relationships between SM experiences and multiple affective states.

However, this study should also be interpreted in light of several limitations. First, findings at the momentary level come from surveys at the same time point, and thus, findings are correlational. However, repeated assessments in the intensive longitudinal design capture high levels of variability between these constructs, improving upon existing research which examines these constructs at one time point or only at the between-person levels. Second, only six affective states were measured throughout the study, and global PA/NA was represented by the sum of lower-order affective states at each prompt. It is possible that other specific emotions may have had differential relationships with SM experiences than the ones examined. Moreover, other computational approaches for PA/NA could have provided information on the variability or affective peaks. Third, survey prompts may have been subject to recall bias, given adolescents may not have necessarily filled them out during SM use and were asked to reflect on most recent use, though EMA provides greater insight into more proximal experiences than other longitudinal approaches. Further, asking adolescents to respond to questions during use may distract from those experiences or influence their affect in the moment. Completion of three daily surveys likely mitigated misremembering of experiences and affect to some extent, but it represents an imperfect measure and approach. Fourth, EMA prompts asking about SM experiences asked about the last time participants used SM versus asking about use since the last prompt. This may mean that, in some instances, participants have double reported on SM use experiences. We believe this is unlikely to apply, given our prior work demonstrates that many adolescents check social media > 100 times per day (ranging into the 1,000 s for some adolescents), but nonetheless may have occurred in our dataset. Fifth, our models include affect and experiences that incorporate emotional responses to SM, which perhaps captures conceptual overlap between the predictors and outcomes. Given these variables only show small-to-moderate associations, they are unlikely to cause a problem of multicollinearity. That said, future studies may choose to test other SM experiences that do not capture emotional responses to probe whether there may be differences from the current study findings. Finally, due to parent study procedures, involving passive smartphone sensing of SM use behaviors, only adolescents who used Android phones were eligible to participate. The inclusion of Android phone users also allowed for recruitment targeting participants at a broad range of socioeconomic status (more reflective of the US population), potentially because Androids come at a broader price point than iPhones, though this study should be replicated within a larger and more representative sample of adolescents.

## Conclusions

This study provided a first step in understanding how, on a momentary level, adolescents’ SM experiences and affect may be related to one another. Positive SM experiences were associated with greater PA and lower NA, while negative SM experiences were associated with lower PA and greater NA. Although findings from this study represent only preliminary evidence, these results underscore associations between SM and affect in the short-term. Given our findings, it appears that the effects of SM experiences are consistent across global affect and corresponding specific emotional states. It is also possible that some SM experiences are associated with specific emotions (e.g., irritability), but that aggregating SM experiences washed out these effects. Future research should investigate this possibility. Taken together, findings from this study provide a useful stepping stone in our understanding of how SM may be associated with both positive and negative mental health outcomes. Using these findings, researchers should continue to build a framework for how developing adolescents may leverage benefits while mitigating the harms of SM.

## Supplementary Information

Below is the link to the electronic supplementary material.

ESM 1 (PDF 137 KB)

## Additional Information

### Data Availability

Data for the current manuscript contains potentially identifiable information and cannot be offered publicly at this time. Deidentified data can be made available upon reasonable request.
