# Supplementary material for: Momentary Associations Between Emotional Responses to Social Media and Affect: Consistency Across Global Affect and Specific Emotional States
Source: Affect Sci. 2024 Aug 22;5(4):417–26. doi: 10.1007/s42761-024-00257-x (PMC11624145; doi:10.1007/s42761-024-00257-x)
Supplement: Supplementary file 1 — (PDF 137 KB) [file 42761_2024_257_MOESM1_ESM.pdf]

Supplementary Table 1. Bivariate correlations including specific ERSM items.

Variable	*M*	*SD*	1	2	3	4	5	6	7	8	9	10	11	12	13	14	15	16
1. Global PA	5.54	3.14																
2. Global NA	3.60	2.91	-.28**															
3. Happy	2.09	1.11	.87**	-.27**														
4. Satisfied	1.74	1.22	.89**	-.30**	.68**													
5. Hopeful	1.72	1.24	.88**	-.17**	.64**	.66**												
6. Worried	1.35	1.26	-.18**	.79**	-.16**	-.23**	-.10**											
7. Irritable	1.07	1.16	-.28**	.78**	-.30**	-.27**	-.18**	.46**										
8. Lonely	1.19	1.30	-.20**	.78**	-.19**	-.21**	-.13**	.39**	.41**									
9. PM SM Social Comparison	1.28	1.29	.02*	.40**	.02	.01	.03*	.29**	.25**	.39**								
10. PC SM Social Comparison	0.00	1.11	-.07**	.16**	-.05**	-.08**	-.04**	.13**	.10**	.15**	.00							
11. PM SM FOMO	1.59	1.28	-.01	.49**	.01	-.06**	.01	.39**	.36**	.40**	.78**	.00						
12. PC SM FOMO	0.00	1.25	-.06**	.15**	-.05**	-.07**	-.03**	.10**	.10**	.15**	.00	.42**	.00					
13. PM SM Nervous	1.04	1.03	.16**	.28**	.13**	.09**	.20**	.23**	.16**	.27**	.70**	.00	.72**	.00				

14. PC SM Nervous	0.00	1.12	-.02	.10**	-.01	-.03*	-.00	.08**	.06**	.09**	-.00	.29**	.00	.29**	.00			
15. PM SM Sad	0.60	0.63	.03*	.28**	.05**	.01	.02	.19**	.17**	.29**	.69**	.00	.65**	.00	.64**	-.00		
16. PC SM Sad	0.00	1.00	-.06**	.15**	-.06**	-.06**	-.05**	.13**	.10**	.11**	.00	.21**	.00	.16**	.00	.28**	.00	
17. PM SM Inspired	1.85	1.18	.36**	.17**	.31**	.29**	.35**	.17**	.13**	.10**	.30**	-.00	.38**	-.00	.33**	-.00	.28**	.00

			17	18	19	20	21
17. PM SM Inspired	1.85	1.18					
18. PC SM Inspired	0.00	1.26	.00				
19. PM SM Supported	2.20	1.32	.88**	.00			
20. PC SM Supported	0.00	1.32	.00	.38**	.00		
21. PM SM Happy	2.06	1.38	.62**	-.00	.82**	.00	
22. PC SM Happy	0.00	1.36	.00	.28**	.00	.51**	-.00

Note. PM: Person Mean (between-person); PC: Person-centered (within-person). SM Social Comparison: ERSM EMA Item 1; SM FOMO: ERSM EMA Item 2; SM Nervous: ERSM EMA Item 3; SM Sad: ERSM EMA Item 4; SM Inspired: ERSM EMA Item 5; SM Supported: ERSM EMA Item 6; SM Happy: ERSM EMA Item 7. * $p < .05$. ** $p < .01$.

Supplementary Material 2. ERSM Full Scale

When you use social media, how often do you…?

Never, Hardly Ever, Sometimes, Often, Always

1. Feel creative because of something you expressed, posted, or shared?
2. Feel proud of yourself because of something you expressed, posted, or shared?
3. **Feel hurt by a negative interaction (e.g., comment, post, DM) with someone else?**
4. Feel connected to your friends?
5. Feel less alone?
6. Feel left out or excluded?
7. **Feel supported and encouraged by your friends?**
8. Feel pressure to show the best version of yourself?
9. **Feel happy because of a positive interaction (e.g., comment, post, DM) with someone else?**
10. Feel like other people are doing better than you?
11. **Feel worried that you are missing out on things?**
12. Feel like you are enjoying yourself and having fun?
13. Feel anxious when you are waiting for someone to respond?
14. Feel pressure to be available to others (e.g., respond right away)?
15. Feel stressed or anxious about the news you see online?
16. Feel comfortable being yourself?
17. Feel upset or bad about yourself after comparing yourself (such as in appearance, accomplishments, relationships, or other things) to other people?
18. Feel happy or good about yourself after comparing yourself (such as in appearance, accomplishments, relationships, or other things) to other people?
19. **Feel that you are not as attractive as other people?**
20. Feel that you are attractive or look good?

21. Feel good about yourself after posting something?
22. Feel disappointed that you did not get enough likes, comments, or shares on your posts?
23. Feel happy that you got likes, comments, or shares on your posts?
24. Feel disappointed that you don’t have enough friends or followers?
25. Feel happy with the number of friends or followers you have?
26. Feel worried that you will not get enough likes, comments, or shares on your posts?
27. Feel bad or disappointed that you did not get enough likes, comments, or shares on something you posted?
28. Feel out of control about how much time you spend on social media?
29. Feel disappointed that you are not friends with or followed by certain people?
30. **Feel afraid to post something because of what other people might think or say?**
31. **Feel excited that you got a lot of likes, comments, or shares on your posts?**
32. Feel upset because of something you saw?
33. Feel happy because of something you saw?
34. Feel accepted for you who are?
35. Feel confident in who you are?
36. Feel uncertain about how others will respond to the things you post on social media?
37. Feel overwhelmed by the number of things I need to look at or respond to on social media?
38. Feel that social media has a NEGATIVE impact on your mood?
39. Feel that social media has a POSITIVE impact on your mood?

Note: Bolded items reflect items that were adapted for EMA usage.

Supplementary Material 3. ERSM EMA Scale

Thinking about the last time you used social media, how much did you feel...

Not at all (0)- Extremely (6)

Negative Experiences
1. That you aren't as good (e.g., attractive/accomplished/etc) or popular as other people?
2. Worried that you were missing out on things?
3. Nervous to post something because of what other people might think or say?
4. Sad or hurt because of a negative interaction (e.g., comment, post, DM) with someone else?

Positive Experiences
5. Inspired by other people or something you saw?
6. Supported or encouraged by others?
7. Happy or excited because of a positive interaction (e.g., comment, post, DM) with someone else?

Supplementary Materials 4. Negative SM Experiences and Global PA/Specific Positive Emotions

	B	*SE*	*p*		*B*	*SE*	*p*
Global Positive Affect				*Satisfaction*			
Person-Mean Negative ERSM	0.09	0.28	.31	Person-Mean Negative ERSM	-0.03	0.10	.77
Person-Centered Negative ERSM	0.03	0.07	.67	Person-Centered Negative ERSM	-0.05	0.03	.06
Happiness				*Hopefulness*			
Person-Mean Negative ERSM	0.02	0.08	.83	Person-Mean Negative ERSM	0.08	0.12	.48
Person-Centered Negative ERSM	0.004	0.03	.89	Person-Centered Negative ERSM	0.01	0.03	.85
